# Waiting for elective general surgery: impact on health related quality of life and psychosocial consequences

**DOI:** 10.1186/1471-2458-7-164

**Published:** 2007-07-19

**Authors:** JP Oudhoff, DRM Timmermans, DL Knol, AB Bijnen, G van der Wal

**Affiliations:** 1 Department of Public and Occupational Health, Institute for Research in Extramural Medicine, Free University Medical Centre, Amsterdam, The Netherlands; 2 Department of Clinical Epidemiology and Biostatistics, Free University Medical Centre, Amsterdam, The Netherlands; 3 Department of Surgery, Medical Centre Alkmaar, Alkmaar, The Netherlands

## Abstract

**Background:**

Long waiting times for elective surgical treatment threaten timely care provision in several countries. The purpose of this study was to assess the impact of waiting for elective general surgery on the quality of life and psychosocial health of patients.

**Methods:**

A cross-sectional questionnaire study with postoperative follow-up was performed among patients on waiting lists for surgical treatment of varicose veins (n = 176), inguinal hernia (n = 201), and gallstones (n = 128) in 27 hospitals.

**Results:**

In each group the waiting period involved worse general health perceptions (GHPQ), more problems in quality of life (EuroQoL), and raised levels of anxiety (STAI) as compared to after surgery (all differences: p < 0.05). Quality of life was not affected in 19–36% of patients.

Emotional reactions to waiting were most negative among patients with gallstones. Prior information about the duration of the wait reduced the negativity of these reactions (p < 0.05).

Social activities were affected in 39% to 48% of the patients and 18%-23% of employed patients reported problems with work during the wait.

Having waited a longer time was associated with worse quality of life among patients with inguinal hernia. Longer waited times also engendered more negative reactions to waiting among patients with inguinal hernia and gallstones (multilevel regression analysis, p < 0.01).

**Conclusion:**

Waiting for general surgery mainly involves a prolonged period of decreased health and an affected psychological and social life of the patient in waiting. Variation in the severity of these consequences across patients indicates that the prioritisation of patients could reduce the overall burden of waiting. Early information about the duration of the delay could further promote a patient's acceptance of waiting.

## Background

As demand of care has outstripped the restricted capacity of public health care services, waiting lists for surgical procedures have become commonplace in many countries [1-5]. In general, waiting lists are comprised of patients whose conditions are not immediately life-threatening and permit treatment to be provided on an elective basis. However, long delays of needed treatment have raised public and political concern, as they could entail diverse negative consequences for patients [6] and subsequently pose a threat to the quality of care. Studies among patients waiting for cardiac or orthopaedic surgery have for instance shown that waiting is associated with deterioration of symptoms [7,8], affected quality of life [8,9] and even death of listed patients [10,11]. Moreover, waiting for treatment is found to cause anxiety, distress and uncertainty among patients [12], and entails prolonged sick leave [13] and loss of income [14,15].

Within general surgery, long waiting times exist for common elective procedures with waits for surgery for varicose veins, inguinal hernia, and gallstones being among the longest. To date, however, little evidence has been gathered on the impact of waiting for treatments of these disorders [16]. Although a small number of studies report on specific physical risks of waiting for these disorders [17-23], the generic burden and psychosocial impact that arises from being on a waiting list is largely unknown. This makes it difficult to assess whether current surgical waiting times are acceptable and warrant a high quality of health care.

This study aims to provide insight into physical and psychosocial impact of waiting for elective surgery for varicose veins, inguinal hernia, and gallstones. In the Netherlands, these three procedures account for a large proportion of patients on waiting lists within general surgery and waiting times are deemed to be long. These patients are put on a waiting list after they first have been referred to surgery by a GP because of the presence of symptoms and after the need for surgery has subsequently been assessed by the surgeon. Once on a waiting list, the patients are normally treated on a first come first served basis. Among the patients waiting for surgery of varicose veins, inguinal hernia, and gallstones, our study looks at (1) the perceived health and quality of life of patients; (2) the levels of generic anxiety and specific negative emotional reactions to waiting; (3) the impact on social functioning; (4) the differences in impact between the three disorders and (5) whether the outcomes on the studied aspects are associated with the duration of the wait.

## Methods

### Participants & procedure

The study was a cross-sectional questionnaire study with postoperative follow-up of the participants. Surgical departments of 27 general hospitals (with over 400 beds each) situated across the Netherlands cooperated. Each hospital was visited between June 2001 and January 2002 and a list was obtained of the adult patients (≥ 18 years) on the waiting list for varicose veins, inguinal hernia, and gallstones. Patients who were scheduled for surgery within two weeks after the visit were excluded for logistical reasons. We also excluded patients if the registration showed that surgery was delayed on the patient's request or if the patient's fitness for surgery was still to be assessed. The following details of eligible patients were recorded: age, sex, and the date of registration on the waiting list. Eligible patients were sent information about the study and an invitation to participate on behalf of a local surgeon. A reminder was sent out after 2 weeks. A preoperative postal questionnaire was dispatched to patients who returned a signed consent form.

To assess the date of surgery of the patients who participated we regularly contacted employees of the participating hospitals during the study. A postoperative questionnaire was sent to each participating patient 90 days after the surgical date.

The study was approved by the VU University Medical Centre medical ethics committee.

### Non response analysis

A non-response analysis was conducted in a selection of 9 participating hospitals. Local hospital employees telephoned the patients who had not returned the consent form and asked the reason for not responding using a short structured questionnaire.

### Measures

#### Patient characteristics

In addition to questions pertaining to demographic details (e.g. age, sex, and working status), participants were asked whether surgery was (to be) provided on an inpatient or outpatient basis, and whether they had been given any indication on the duration of the wait when they were placed on the waiting list. The latter was asked because anecdotal evidence suggested that different hospitals or individual surgeons (if waiting lists were maintained on the level of individual surgeons) have different policies with regard to planning surgery and/or about providing estimates about the expected length of the wait.

#### General health perceptions and quality of life

To measure the perceived health of patients we used the Dutch version of the RAND General Health Perceptions Questionnaire (GHPQ) [24]. The GHPQ consisted of 24 statements on general health perceptions such as 'My health is excellent'. Participants were asked to indicate the degree to which each statement applied to their situation on a 5 point scale ranging from 'definitely true' to 'definitely not true'. The answers add up to an overall score ranging from 24 to 120, with higher scores indicating better perceived health.

To assess the experienced problems in the participant's health related quality of life we administered the EQ-5D descriptive questionnaire from the EuroQol group [25]. The EQ-5D addressed 5 dimensions of quality of life: pain/discomfort, mobility, anxiety/depression, usual activities, and self-care. On each dimension, participants were to indicate whether they experienced 'no problems', 'some or moderate problems', or 'extreme problems'.

#### Psychological consequences

We measured the generic level of anxiety among patients using the state anxiety scale from the Dutch version of the State-Trait Anxiety Inventory (STAI) [26,27]. This scale consists of 20 statements that address the individual's situational anxiety. Participants are to indicate the degree to which each statement reflects their current feelings on a 4 point scale. The responses add up to a score between 20 and 80, with higher scores indicating higher levels of state anxiety.

To assess the degree to which participants had specific negative emotional reactions to waiting, we constructed a scale consisting of 7 items such as "The wait for surgery makes me nervous". Participants were asked to indicate the level of agreement with each item on a 5 point Likert scale (assigned values: 0 to 4). Summing of the responses produced a scale score ranging from 0 to 28, with higher scores indicating more negative reactions to waiting (Cronbach's α = 0.89). An overall score of 14 reflected that responses were on average 'neutral'. The items and response categories are shown in appendix A.

#### Social consequences

To asses the consequences of waiting on social aspects we asked the participants during the wait whether their condition interfered with leisure activities (meeting family or friends, going out, and practising sports or hobbies); with caring for dependants; and with their daily work.

#### Waiting time intervals

Throughout the study we used the following definitions of waiting time intervals:

- '*total waiting time'*: the number of days between the date of registration on the waiting list and the date of surgery.

- *'waited time'*: the number of days between registration on the waiting list and completion of the preoperative questionnaire.

### Analysis

First, we used descriptive statistics to analyse the responses of the participants. Where appropriate, differences between patient groups and between the pre- and postoperative results were assessed using t-tests and nonparametric tests depending on the type of data (see results section). All descriptive analyses were performed using SPSS 12.0.1 [28], with p < 0.05 taken as statistically significant.

Second, we performed multilevel regression analysis on the diverse outcome measures to assess the effect of the time patients had waited before completing the preoperative questionnaire. We allowed for potential effects arising from differences between the hospitals where the patients were registered (for instance, in terms of waiting list management or used indications for surgery). Multilevel linear regression analysis (general linear mixed model: procedure Mixed in SPSS 12.0.1 [28]) was performed on the scores for general health perceptions (GHPQ), for state anxiety (STAI), and for the negative emotional reactions to waiting. In the latter analysis we also assessed whether being given information about the duration of the wait (i.e., a planned date of surgery, or a surgical date by approximation) had an effect on a patient's emotional reactions to waiting.

Multilevel logistic regression was applied to assess whether the length of time waited was associated with reporting problems on each dimension of quality of life (EQ-5D), with experiencing impairments in leisure activities, or on work. These analyses were performed using MLwiN 2.0 [29].

The implicit prioritisation of patients with more severe problems could mask a possible positive association between the time patients had waited and the severity of problems, in terms of regression analyses. In order to suppress this masking effect we included the total waiting time as a variable in the analysis. As a consequence, the found effect for the waited time is applicable when the total waiting time remains constant in the regression equation. We corrected for differences related to age, sex, and treatment on an in- or outpatient basis if significant in all the analyses carried out. As the distributions of the waited times and the total waiting times were skewed we also applied a logarithmic transformation on these variables to approach normality.

As the number of regression analyses undertaken was relatively large, we took only p < 0.01 as statistically significant.

In addition to all analyses we investigated whether there were differences between participants who returned the postoperative questionnaire and those who were lost to follow up or did not return the postoperative questionnaire, to see if there might have been selective drop-out. This analysis focused the responses for EQ-5D as it was a generic outcome measure covering multiple dimensions.

## Results

### Participants

Table 1 shows the number of eligible patients, the response rates, and reasons for drop out throughout the study. Pre- and postoperative questionnaires were completed respectively by 176 and 134 patients with varicose veins, 201 and 152 inguinal hernia patients, and 128 and 98 patients with gallstones. Table 2 gives the characteristics of the participants. The respective median waiting times for surgery per disorder were 170, 115 and 111 days. In each group, a majority of participants (50–63%) reported that no indication on the duration of the wait was given when they were placed on the waiting list. An exact date of planned surgery was given to 11–19% of the participants.

**Table 1:** Eligible patients, response rates, and reasons for drop out throughout the study

	Varicose veins			Inguinal hernia			Gallstones		
	n	%	(%)	n	%	(%)	n	%	(%)
Eligible	667	100		748	100		512	100	
Informed consent	270	40.5		342	45.7		207	40.4	
**Preoperative response (valid)**	**176**	26.4	***(100)***	**201**	26.9	***(100)***	**128**	25.0	***(100)***
									
Surgery not in original hospital	11		*(6.3)*	20		*(10.0)*	9		*(7.0)*
Postoperative q. not returned	31		*(15.3)*	29		*(11.9)*	21		*(10.9)*
**Postoperative response (valid)**	**134**		***(76.1)***	**152**		***(75.6)***	**98**		***(76.6)***

q., questionnaire

**Table 2:** Characteristics of the participants and the information they received about the surgical date when placed on the waiting list

	Varicose veins		Inguinal hernia		Gallstones	
	Preoperative n = 176	Postoperative n = 134	Preoperative n = 201	Postoperative n = 152	Preoperative n = 128	Postoperative n = 98
Age in years, mean (SD)	51.6 (11.9)	52.0 (11.7)	62.9 (11.4)	63.0 (11.1)	52.6 (14.4)	52.9 (14.5)
Sex, % male	31.8	33.6	96.5	96.1	35.2	35.7
Total waiting time in days, median	170	166	115	113	111	98
Surgery on inpatient basis, %	39.7	42.9	62.9	72.8	94.4	96.9
No information on surgical date, %	51.2		50.0		62.5	
Surgical date by approximation, %	29.2		36.8		26.7	
Exact date of planned surgery, %	19.6		13.2		10.8	

A comparison of the study participants with the eligible patients who did not participate showed similar distributions of sex, and waited times, but a higher mean age both among participants for varicose veins (52 vs. 48 years; t_665 _= 3.59, p < 0.001), and among the participants for inguinal hernia (63 vs. 57; t_746 _= 4.85, p < 0.001) (data not shown).

The non-response analysis among 230 (19% to 23% per disorder) of the patients who did not consent to participate showed the following results: 13% did not consent because they had no problems with being on the waiting list, 8% because surgery would take place soon and 6% because they were tired of waiting. Seventeen percent stated that they considered the study not applicable to them as surgery had already taken place (elsewhere) or the delay was owed to medical or personal reasons. The remaining 56% reported miscellaneous reasons not related to being on a waiting list (predominantly: 'lack of time' or 'forgotten').

### General health perceptions and quality of life

In all three groups, the scores for general health perceptions (GHPQ) were significantly lower, i.e. worse, during the wait than after surgery (Table 3). The largest improvement in perceived health was found for patients with gallstones, whose preoperative scores were significantly lower than the scores for varicose veins(t_297 _= 4.04, p < 0.001) and inguinal hernia(t_321 _= 3.27, p = 0.001). Multilevel linear regression analyses showed no association between the time patients had waited and the scores for general health perceptions (Table 3).

**Table 3:** Pre- and postoperative scores for general health perceptions (GHPQ) and the associations with waiting time

	Varicose veins			Inguinal hernia			Gallstones		
	Preoperative n = 175		Postoperative n = 134	Preoperative n = 199		Postoperative n = 150	Preoperative n = 124		Postoperative n = 97
Mean	85.0		87.3*	83.7		87.4*	78.2^a,b^		86.5*
SD	14.2		15.5	14.8		15.3	14.5		13.8

ML linear regression**	b	p		b	p		b	p	
log_e_(waited time)	2.00	0.29		-2.74	0.21		-2.12	0.46	
log_e_(total waiting time)	-0.60	0.79		1.59	0.51		0.45	0.88	

SD, standard deviation; ML, multilevel; p, p-value

* Significant difference between pre- and postoperative scores (Paired t-test, p < 0.05).

**The results of the regression analysis were corrected for age, sex, and an in- or outpatient basis of surgery if significant.

a significant difference between varicose veins and gallstones(t-test, p < 0.05; (no differences in postoperative scores)

b significant difference between inguinal hernia and gallstones(t-test, p < 0.05; (no differences in postoperative scores)

Overall improvement by surgery was also found for the participants' health related quality of life (EQ-5D, Table 4). During the wait respectively 139 of 171 (81%) patients with varicose veins, 125 of 194 (64%) patients with inguinal hernia, and 88 of 121 (74%) patients with gallstones reported having problems on one or more dimension of quality of life. After surgery these numbers had decreased significantly to: 71 of 128 (55%) for varicose veins (McNemar test: χ^2 ^= 19.15, df = 1, p < 0.001); 53 of 146 (36%) for inguinal hernia (χ^2 ^= 22.22, df = 1, p < 0.001), and 41 of 95 (43%) for gallstones (χ^2 ^= 16.53, df = 1, p < 0.001). Each condition was found to involve problems on the dimensions for pain/discomfort, and usual activities during the wait. Moreover, the patients with inguinal hernia reported significantly more often problems with mobility during the wait than after surgery, whereas surgery involved a significant improvement on the dimension for anxiety/depression among patients with gallstones (Table 4). A comparison between the preoperative responses of participants who did return the postoperative questionnaire and of those who did not, showed no differences for patients with varicose veins, whereas differences (Chi-square, p < 0.05) on respectively one (self-care) and three (mobility, self-care, anxiety) dimensions were found for patients with inguinal hernia and gallstones. In both groups, those who did not return the postoperative questionnaire reported a problem on these dimensions slightly more often than those who returned the questionnaire.

**Table 4:** Participants (%) reporting problems in quality of life (EQ-5D) during the wait and 3 months after surgery

	Varicose veins		Inguinal hernia		Gallstones	
	Preoperative n = 174	Postoperative n = 134	Preoperative n = 198	Postoperative n = 150	Preoperative n = 127	Postoperative n = 97
**Pain/Discomfort**						
none	24.3	50.4*	43.9^a^	70.7^a ^*	35.7^b^	63.9^b ^*
moderate	73.4	48.1	52.0	28.7	54.8	35.1
extreme	2.3	1.5	4.1	0.7	9.5	1.0
						
**Mobility**						
no problems	63.0	74.6	62.6	89.4^a ^*	82.9^b,c^	83.5
some problems	36.4	25.4	37.4	10.6	16.3	16.5
confined to bed	0.6	0	0	0	0.8	0
						
**Anxiety/depression**						
none	88.5	87.0	90.9	92.6	66.9^b,c^	88.5*
moderate	9.8	13.0	8.6	7.4	28.3	11.5
extreme	1.7	0	0.5	0	4.7	0
						
**Usual activities**						
no problems	59.8	81.2*	53.5	88.6*	66.1^c^	81.4*
some problems	40.2	18.0	44.4	11.4	30.7	18.6
unable to	0	0.8	2.0	0	3.1	0
						
**Self-care**						
no problems	99.4	97.7	97.5	95.9	97.6	99.0
some problems	0.6	2.3	2.5	4.1	2.4	1.0
unable to	0	0	0	0	0	0

* significant difference between the pre- and postoperative rate of reporting problems within the disorder (McNemar test, p < 0.05).

a significant difference between varicose veins and inguinal hernia(χ^2^-test, p < 0.05).

b significant difference between varicose veins and gallstones(χ^2^-test, p < 0.05).

c significant difference between inguinal hernia and gallstones(χ^2^-test, p < 0.05).

For varicose veins and gallstones, multilevel logistic regression analysis showed no relationship between the time patients had waited and the reporting of problems with quality of life (Table 5). For inguinal hernia it was found that patients who had waited longer had a significantly (p < 0.01) higher probability of reporting problems with pain/discomfort, mobility, and usual activities. However, it was found that inguinal hernia patients who reported problems with pain/discomfort and mobility were also more likely to experience shorter total waiting times for surgery.

**Table 5:** The associations* between the time spent waiting and the likelihood of reporting a problem with quality of life (EQ-5D)**

	Varicose veins n = 174		Inguinal hernia n = 199		Gallstones n = 126	
	OR	95% CI	OR	95% CI	OR	95% CI
**Pain/discomfort**						
log_e_(waited time)	1.527	0.843–2.765	3.370	1.567–7.250	0.763	0.305–1.910
*log_*e*_(total waiting time)*	*0.487*	*0.238–0.997*	*0.141*	*0.057–0.344*	*0.952*	*0.349–2.596*
						
**Mobility**						
log_e_(waited time)	0.715	0.405–1.265	2.878	1.320–6.271	1.357	0.204–9.016
*log_*e*_(total waiting time)*	*1.179*	*0.602–2.309*	*0.195*	*0.08–0.470*	*0.389*	*0.053–2.869*
						
**Anxiety/depression**						
log_e_(waited time)	1.002	0.422–2.380	5.228	0.975–28.02	1.639	0.591–4.54
*log_*e*_(total waiting time)*	*1.770*	*0.596–5.259*	*0.135*	*0.02–0.821*	*0.589*	*0.200–1.728*
						
**Usual activities**						
log_e_(waited time)	0.847	0.485–1.478	2.499	1.305–4.787	1.332	0.488–3.640
*log*_*e*_*(total waiting time)*	*0.982*	*0.508–1.898*	*0.327*	*0.158–0.676*	*0.698*	*0.237–2.056*

OR, Odds Ratio; CI, Confidence Interval

*The results of the multilevel regression analysis were corrected for age, sex, and an in- or outpatient basis of surgery if significant.

**For analysis, the responses for category 1 ('some problems'/'moderate') and category 2 ('extreme' or 'unable to') were combined to indicate 'a problem' as opposed to response category 0 ('no problem'). This was done because the number of participants in response category 2 was too small to analyse separately. The effect of waiting on problems with self care was not analysed as the number of patients with problems on this dimension was too small

### Psychological consequences

In all three groups, the participants' mean levels of generic anxiety (STAI) were significantly higher during the wait than at 3 months after surgery (Table 6). The preoperative anxiety scores were highest for patients with gallstones, but their mean score was only slightly and not significantly higher than the mean score obtained from a sample of the general population [26] (40.56 vs. 38.33; t_123 _= 1.964, p = 0.054).

**Table 6:** Pre- and postoperative scores for state anxiety (STAI) and their associations with waiting time

	Varicose veins			Inguinal hernia			Gallstones		
	Preoperative n = 175		Postoperative n = 133	Preoperative n = 200		Postoperative n = 149	Preoperative n = 124		Postoperative n = 95
Mean	36.3		35.4*	35.4		32.3^a^*	40.6^b,c^		34.8*
SD	9.6		9.4	10.0		8.6	12.7		11.0
ML linear regression**	b	p		b	p		b	p	
log_e_(waited time)	-1.88	0.15		3.54	0.012		2.73	0.27	
*log*_*e*_*(total waiting time)*	*0.16*	*0.92*		*-4.06*	*0.010*		*-2.22*	*0.41*	

SD, standard deviation; ML, multilevel,;p, p-value

* significant difference between pre- and postoperative scores (paired t-test, p < 0.05).

**The results of the multilevel regression analysis were corrected for age, sex, and an in- or outpatient basis of surgery if significant.

a significant difference between varicose veins and inguinal hernia (t-test, p < 0.05).

b significant difference between varicose veins and gallstones (t-test, p < 0.05).

c significant difference between inguinal hernia and gallstones (t-test, p < 0.05).

Multilevel linear regression analysis showed a trend towards higher anxiety scores with longer waited times (p = 0.012) among the inguinal hernia patients (Table 6).

Similar to the results for generic anxiety, the scores on the scale for negative emotional reactions to waiting were highest among the patients with gallstones (Table 7). In fact, only patients with gallstones had a mean score that exceeded the level of 14 points that reflected on average neutral emotional reactions to waiting (t_126 _= 6.159, p < 0.001).

**Table 7:** Scores for negative emotional reactions to waiting and their associations with waiting times and information about the expected duration of the wait

	Varicose veins		Inguinal hernia		Gallstones	
	n = 175		n = 199		n = 127	
Mean	12.9		13.6		17.8^a,b^	
SD	7.8		7.7		6.9	
ML linear regression*	b	p	b	p	b	p
log_e_(waited time)	-1.07	0.30	3.17	0.004	5.00	0.003
*log*_*e*_*(total waiting time)*	*0.46*	*0.74*	*-4.03*	*0.001*	*-5.07*	*0.006*
Surgical date by approximation	-3.37	0.02	-2.53	0.04	-0.87	0.56
Exact date of planned surgery	-4.34	0.03	-6.69	<0.001	-3.57	0.09

SD, standard deviation; ML, multilevel; p, p-value

*The results of the multilevel regression analysis were corrected for age, sex, and an in- or outpatient basis of surgery if significant.

a significant difference between varicose veins and gallstones (t-test, p < 0.05).

b significant difference between inguinal hernia and gallstones (t-test, p < 0.05).

Multilevel linear regression analyses showed that the emotional reactions to waiting tended to be less negative when patients received information on the date of surgery when they were placed on the waiting list (Table 7). A significant reduction (p < 0.01) was, however, only found for patients with inguinal hernia who were given the exact date of surgery. Among patients with inguinal hernia and patients with gallstones the emotional reactions to waiting were found to be significantly more negative when patients had waited longer.

### Social consequences

In all, 68 of 174 (39%) patients with varicose veins, 97 of 200 (48%) patients with inguinal hernia, and 49 of 126 (39%) patients with gallstones reported that their condition interfered with leisure activities during the wait. Varicose veins and inguinal hernia especially interfered with performing sports and hobbies (35% and 48% of patients with varicose veins and inguinal hernia respectively) whilst limitiations in meeting family/friends or going out were reported less frequently (respectively 9% and 17% among patients with varicose veins and 10% and 16% of patients with inguinal hernia). Thirty percent of patients with gallstones reported limitations in going out, and respectively 20% and 22% reported interferences with hobbies and meeting family or friends. In all three groups there was no association between the time spent waiting and reporting a limitation in leisure activities (for varicose veins: OR = 0.770, 95% CI = 0.421–1.406; for inguinal hernia: OR = 0.931; 95% CI = 0.509–1.704; for gallstones: OR = 0.936, 95% CI = 0.393–2.228).

Of 58 patients with varicose veins who reported to take care of dependants, 4 (6.9%) reported that they were limited in doing so during the wait owing to their condition. For inguinal hernia and gallstones this was reported by respectively 1 of 31 (3.2%) patients and 5 of 51 (9.8%) patients.

Employment rates among the participants were respectively: 32% (65 of 201 patients) patients with inguinal hernia, 44% (55 of 125 patients) patients with gallstones and 53% (92 of 175) patients with varicose veins. Of these employed participants, 18% to 23% reported that their condition interfered with work during the wait. Sick leave was predominantly reported by patients with gallstones (7 of 55 patients (12%)), whereas sick leave occurred less among patients with varicose veins (2 of 92 patients (2.2%)), and inguinal hernia (2 of 65 patients (3.1%)). The latter two groups mostly reported that they were either unable to perform certain specific tasks or that some adjustments to the working place were needed (14 of 92 patients with varicose veins (17%); 13 of 65 patients with inguinal hernia (20%)). Logistic regressions analysis did not show that the time spent waiting was associated with whether patients reported difficulties in work or not (for varicose veins: OR = 1.786, 95% CI = 0.513–6.221; for inguinal hernia: OR = 1.611, 95% CI = 0.430–6.032; for gallstones: OR = 2.691, 95% CI = 0.289–25.103).

## Discussion

Insight into the consequences of waiting for treatment provides a means to evaluate the acceptability of waiting times and their impact on the quality of health services [30]. Our results show that waiting times for surgery for varicose veins, inguinal hernia, and gallstones entail that patients are temporarily withheld significantly beneficial effects of surgery and commonly experience a period of decreased perceived health, problems in quality of life, raised anxiety, and limitations in social life. Yet, the extent of these physical, psychological, and social consequences of waiting varies between patients which can partly be attributed to the specific disorder. A relevant indicator in this respect may be the emotional reactions to waiting, which are most negative in the patients with gallstones whereas a more neutral attitude was averagely found in patients with varicose veins, and inguinal hernia. The latter conditions correspondingly had less impact than gallstones on the perceived health and anxiety levels of patients. This match between the health impact of the condition and the attitude towards waiting reflects the finding by others that a patient's symptoms are an important determinant of the acceptance of the delay [31]. This acceptance seems to be further promoted by clarity over the duration of the wait, given the found reduction in the negative reactions to waiting when patients knew the surgical date in advance.

Next to the differences in the impact between the specific conditions, the difficulties that patients experience during the wait vary to an important extent within each disorder. Whereas in each group 19% to 36% of the patients have no problems on any dimension of quality of life, the condition interferes with normal social activities in a substantial number of patients and affects working capacity in yet a smaller group, including sick leave in 12% of patients with gallstones. Rather similar differences in the impact of waiting between patients with these disorders are also found for the condition-specific symptoms [32].

Although our overall findings thus seem to justify that patients with the studied conditions generally receive a relatively low urgency, waiting clearly seems not equally acceptable for all patients. To some extent, recognition of this may be naturally present as overall waiting times were shortest for patients with gallstones, and as having more problems was associated with shorter waiting times for patients with inguinal hernia. The findings do however raise questions about the appropriateness of the fairly common policy to treat patients on a first come first served basis as was present at the time of our study but is still the predominant policy at this moment. Rather, a more explicit and extensive prioritisation of patients on waiting lists, as has been debated or issued in some countries [33-36], could likely further reduce the overall burden of waiting for these conditions.

In addition to the condition's health impact at a given moment, the acceptability of treatment delays depends on what might happen during the wait. Our results indicate that waiting times as in our study do not involve significant gradual worsening of the perceived health for patients with varicose veins or gallstones, although the latter group did show increasingly more negative reactions towards the wait itself. Among inguinal hernia patients, however, we found that a longer time spent waiting was associated with a higher probability of reporting problems in health. This finding likely suggests that deterioration of health while waiting indeed occurs among inguinal hernia patients, although we could not accurately assess the extent and degree of it owing to the cross-sectional design of our study. Our results do, however, correspond with the finding by others that the likeliness of developing pain from an inguinal hernia increases with time [37].

Apart from the possibility of gradual worsening of health, delayed surgery for inguinal hernia or gallstones puts patients at risk for developing acute complications which require hospital admission and urgent treatment. The literature on this topic suggests that this risk particularly applies to patients waiting for cholecystectomy. While strangulation of inguinal hernia seems uncommon among patients on waiting lists [38-40], gallstone-related complications are observed more frequently[19-23,41] as emergency admission rates of up to 0.9 per 100 patients per week are reported [22]. Although the impact of waiting will thus for most patients be indicated by the severity of their condition at initial presentation, assessment of the acceptability of waiting for patients with inguinal hernia and gallstones should include an estimation of the likeliness of worsening of health and the identification of risk factors for complications.

### Limitations

Although the number of participants in each group are sufficient for the objectives of our study, the response rates are disappointing. The results of the non response analysis do not indicate that reluctance to participate was owed to a specific reason related with being on a waiting list. Yet, as the study was performed at a time when waiting lists throughout health care received high media coverage and were generally portrayed as a failure from the government to provide timely care, patients might have thought efforts should be directed at reducing general waiting times rather than studying their consequences. This in addition to the relatively non-urgent nature of the conditions might have contributed to the low response rates. Our samples did appear representative with regard to waiting times and sex distributions of patients, but over-represent those of slightly higher age for varicose veins and inguinal hernia. Our results thus seem representative for patients on the waiting lists, but the difference with regard to age of the responders may have entailed a slight under representation of employed patients.

The analyses that looked at differences between those who returned the postoperative questionnaire and those who did not, did not suggest that there was obvious selective drop-out among patients with varicose veins or inguinal hernia. The patients with gallstones who dropped out or were lost to follow up, however, seemed to have worse health on some dimensions of quality of life than to those who returned the postoperative questionnaire. Whereas this does not affect the results of our comparison of pre- and postoperative scores (since these were paired comparisons), it might suggest that the postoperative results on gallstones were not entirely representative for patients who were relatively worse off. However, remarkably, the dimensions on quality of life that those who dropped out had lower scores for, did not seem to improve after surgery among those who did participate. This could suggest that the worse health might have been associated to another disorder than gallstones and that this was a reason for not participating in the postoperative part of the study.

We used a cross-sectional design in order to provide relevant insight into the health problems of patients on surgical waiting lists. Owing to the design, we estimated the effect of waiting indirectly by means of regression analyses on the responses of different participants which leave it indistinct which effect waiting had on the health of individual patients. Additionally, we used a self-made scale to measure the negative emotional reactions to waiting. Although the scale showed to have good reliability, it is not fully validated. The results regarding the negative emotions to waiting should thus be taken with care.

## Conclusion

Long waiting lists for elective treatment pose a challenge to the quality of public health care services with which patients, doctors, and responsible health authorities have to deal. Our findings show that the consequences of waiting for surgery vary substantially between patients which to some extent relates to the specific condition. While relatively long waits seem justifiable for a considerable group of patients, this is less obvious for patients who experience difficulties while waiting or are at risk for serious complications. It seems therefore important for doctors to assess the anticipated burden of waiting at the patients' first consultation and prioritise the patients correspondingly. Whereas it additionally seems appropriate to inform patients on the likeliness of deterioration of health and the risk of complications during the wait, early information about the expected duration of the delay could further enhance a patient's acceptance of waiting.

## Competing interests

The author(s) declare that they have no competing interests.

## Authors' contributions

JO contributed to data collection, analysis and interpretation of the data and drafted the manuscript. DT contributed to study design, and analysis and interpretation of the results helped to draft the manuscript. DK contributed to analysis and interpretation of data helped to draft the manuscript. AB contributed to study design, and the interpretation of the data helped to draft the manuscript. GvdW contributed to the study design and the interpretation of the data helped to draft the manuscript. All authors read and approved the final manuscript.

## Appendix A

Items and response categories of the scale for the negative emotional reactions to waiting

### Items

- The wait for surgery is highly annoying.

- The wait for surgery makes me nervous

- The quicker I receive surgery, the better I will feel.

- The wait for surgery makes me worry.

- I find it very unpleasant that I am required to wait for surgery.

- I doesn't matter to me that I have to wait for surgery.

- It irritates me every day that I still have not received surgery

### Response categories

Disagree completely; Disagree to some extent; Neutral; Agree to some extent; Agree completely.

## Pre-publication history

The pre-publication history for this paper can be accessed here:


